# The Potential of Natural Oils to Improve Inflammatory Bowel Disease

**DOI:** 10.3390/nu15112606

**Published:** 2023-06-01

**Authors:** Yaxi Zhou, Diandian Wang, Hao Duan, Shiqi Zhou, Jinhong Guo, Wenjie Yan

**Affiliations:** 1 College of Biochemical Engineering, Beijing Union University, No. 18, Xili District 3, Fatou, Beijing 100023, China; 2 Beijing Key Laboratory of Bioactive Substances and Functional Food, College of Biochemical Engineering, Beijing Union University, 197 North Tucheng West Road, Beijing 100023, China

**Keywords:** natural oil, inflammatory bowel disease, therapeutic drugs, mechanisms of action

## Abstract

Inflammatory bowel disease (IBD) is a chronic intestinal inflammatory disorder that includes ulcerative colitis (UC) and Crohn’s disease (CD), the exact cause of which is still unknown. Numerous studies have confirmed that diet is one of the major environmental factors associated with IBD, as it can regulate the gut microbiota and reduce inflammation and oxidative stress. Since the consumption of oil is essential in the diet, improving IBD through oil has potential. In this article, we first briefly reviewed the current treatment methods for IBD and introduce the role of natural oils in improving inflammatory diseases. We then focused on the recent discovery of the role of natural oils in the prevention and treatment of IBD and summarized their main mechanisms of action. The results showed that the anti-inflammatory activity of oils derived from different plants and animals has been validated in various experimental animal models. These oils are capable of improving the intestinal homeostasis in IBD animal models through multiple mechanisms, including modulation of the gut microbiota, protection of the intestinal barrier, reduction in colonic inflammation, improvement in oxidative stress levels in the intestine, and regulation of immune homeostasis. Therefore, dietary or topical use of natural oils may have potential therapeutic effects on IBD. However, currently, only a few clinical trials support the aforementioned conclusions. This review emphasized the positive effects of natural oils on IBD and encouraged more clinical trials to provide more reliable evidence on the improvement of human IBD by natural oils as functional substances.

## 1. Introduction

Inflammatory bowel disease (IBD) encompasses two conditions, ulcerative colitis (UC) and Crohn’s disease (CD), which were first discovered in 1859 and 1932, respectively. Therefore, they are considered relatively new diseases [1,2]. UC and CD are chronic inflammatory bowel diseases that usually cause progressive weakness and cannot be fully cured. UC lesions are mostly located in the rectum and colon, and symptoms include abdominal pain, bloody diarrhea, and weight loss. UC can occur at any age, and it is difficult to treat, with the risk of developing colon cancer, which severely affects human health [3]. CD can occur in any part of the gastrointestinal tract, mainly manifested as abdominal pain, diarrhea, and intestinal obstruction. CD has the characteristics of having a long course and showing recurrent episodes [4]. In recent years, the number of IBD patients has increased, especially in Asia [5]. As of 2017, the reported cases of IBD patients worldwide reached 6.8 million [6]. In the past few decades, the incidence and prevalence of IBD have been generally higher in developed countries than in developing countries. However, in recent years, the incidence of IBD has been rapidly increasing and continuing to rise in certain Asian regions, such as China, Japan, and Korea [7,8].

The pathogenesis of IBD is very complex and is usually the result of the combined action of multiple factors. Studies have shown that the causes of IBD are related to environmental factors such as diet, smoking, and environmental hygiene, as well as immune system disorders, gut microbiota imbalances, psychological factors, and genetics [4,9,10]. The recurrent causes make the treatment of IBD more difficult, so there is currently no single therapeutic drug or method that can completely cure all types of IBD patients.

In recent years, many studies have found that many natural compounds have a certain preventive, improvement, and therapeutic effect on IBD. For example, natural flavonoids, terpenes, glycosides, polyphenols, quinones, alkaloids, and coumarin compounds have shown certain IBD treatment activities [11]. In addition, more research has focused on the IBD treatment activity of natural polysaccharides. Because some natural polysaccharides have significant physiological activities such as the anti-inflammatory, immune regulation, and antioxidant effects, and they have a wide range of sources, good safety, and few side effects, with broad prospects in improving IBD [3,12]. Furthermore, some natural proteins and bioactive peptides have been found to have enormous potential to improve IBD [13,14]. Moreover, more and more studies have revealed the IBD treatment activity of some natural oils, such as olive oil, perilla oil, garlic oil, flaxseed oil, walnut oil, emu oil, and so on. The anti-IBD activity of these natural oils from different plants and animals has been verified in a large number of experimental IBD animal models and some clinical trials. However, there is currently no review article that elaborates on the therapeutic effects of natural oils for IBD. Therefore, in this article, we focus on the role of natural oils discovered in recent years in the prevention and treatment of IBD and summarize their main mechanisms of action.

## 2. Current IBD Treatment Drugs and Methods

Currently, the main treatment for IBD is still drug therapy, with other supplementary treatment methods. The five main categories of drugs used for IBD treatment include aminosalicylic acid, corticosteroids, immunosuppressants, biologics, and microbial agents [3]. Aminosalicylic acid drugs such as sulfasalazine, mesalazine, and olsalazine are commonly used, with mesalazine being a first-line drug for treating mild to moderate IBD. It works by inhibiting the NF-κB pathway and scavenging free radicals to relieve inflammation [15,16]. Aminosalicylic acids are effective in treating most patients with few reported adverse reactions, but long-term use can have side effects, such as myocarditis [17]. Although low-dose aminosalicylic acids have low toxicity and a low risk of dose-dependent toxicity, their effectiveness is not as good as high-dose treatment. Therefore, appropriate drug doses should be chosen based on the severity of the disease when administering these drugs. Representative corticosteroids include prednisolone, methyl-prednisolone, hydrocortisone, budesonide, budesonide MMX, and beclomethasone dipropionate [18]. Due to their strong drug dependence and certain side effects, corticosteroids cannot be used for long periods and are currently only used for IBD patients who are unresponsive to aminosalicylates [19,20]. Similarly, some immunosuppressive agents are also used to treat IBD patients who are unresponsive to aminosalicylates. The currently used immunosuppressive agents include thiopurines, methotrexate, and cyclosporine [21]. Immunosuppressive agents can relieve the drug dependence of corticosteroids in IBD patients and have a relieving effect on severe IBD patients, especially those with acute severe UC [22,23].

In addition, when the above-mentioned drug treatments are ineffective, some biological agents are also used to treat IBD. Currently, there are five categories of biological agents used to treat IBD, including TNF-α inhibitors such as infliximab, adalimumab, and golimumab; anti-adhesion molecules such as natalizumab and vedolizumab; anti-interleukin drugs such as ustekinumab, risankizumab, and brazikumab; Janus kinase inhibitors such as tofacitinib, filgotinib, and upadacitinib; and Sphingosine 1 Phosphate Receptor Modulators such as etrasimod and ozanimod [24,25]. These biological agents are not suitable for the oral administration due to their high sensitivity to the gastrointestinal environment, and they are not applicable for the treatment of all IBD patients [26]. Some microbial agents such as probiotics, such as *Escherichia coli* Nissle 1917 and VSL#3, can be used for the relief and treatment of moderate to severe IBD because they are associated with intestinal microbiota [27].

**Figure 1:**
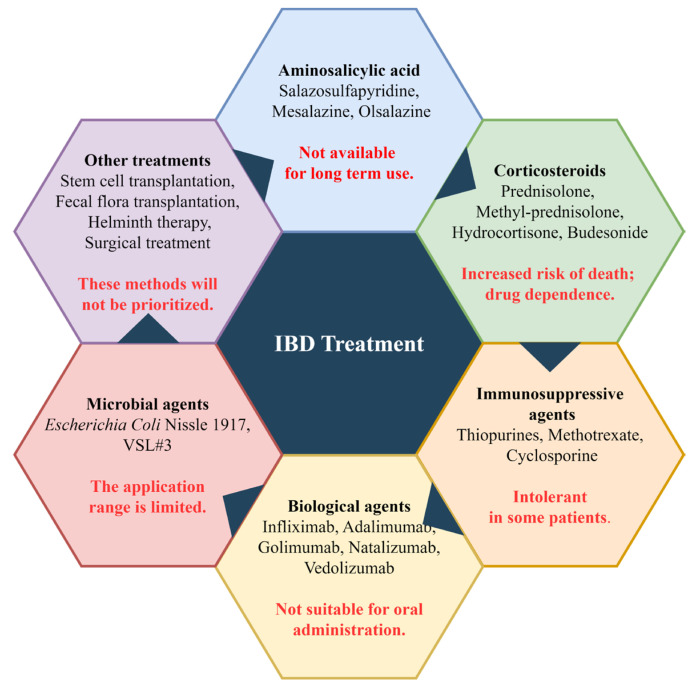
Current therapeutic drugs and methods for IBD. The content highlighted in red font indicates the limitations of the method.

In addition to drug therapy, some treatment methods such as stem cell transplantation, fecal microbiota transplantation, helminth therapy, and surgical treatment are also used to treat IBD. These methods are usually used when drug therapy is ineffective. Figure 1 demonstrates the current drugs and methods of treatment for IBD. Stem cell transplantation therapy is considered a relatively safe treatment, but because it is a relatively new IBD treatment method, more clinical trials are needed to determine its therapeutic efficacy and safety [28,29]. Fecal microbiota transplantation is safe for the treatment of IBD and has a good therapeutic effect for IBD patients with *Clostridium difficile* infection [30]. Helminth therapy can also improve clinical symptoms in some IBD patients, but its therapeutic mechanism is unclear and very complex, so it is generally not used [31]. In cases of particularly severe IBD and when the above methods are ineffective, surgical treatment such as total proctocolectomy may be selected [32].

## 3. The Anti-Inflammatory Effects of Natural Oils

Natural oils have unique biochemical and therapeutic functions, which give them enormous potential in the treatment of diseases and functional food areas. Some functional oils have been proven to have anti-inflammatory, antioxidant, and immune-modulatory functional activities. For example, *Cabernet sauvignon* grape seed oil has strong anti-inflammatory and antioxidant functions due to its high content of unsaturated fatty acids and endogenous antioxidants (tocopherols, tocotrienols, and phenolic compounds). It can regulate lipid metabolism in mice with hyperlipidemia and improve inflammation and oxidative stress in mice [33].

Bacterial lipopolysaccharides (LPS) are widely used to induce inflammation models [34]. Verification of the anti-inflammatory activity of a compound is often carried out by using LPS-induced cell or animal inflammation models. Some studies have found that natural oils have good anti-inflammatory effects on LPS-induced inflammation models. One study found that continuous supplementation with argan and olive oils for 25 days significantly protected against LPS-induced septic shock in mice, which was related to improvement in mouse liver oxidative stress and inflammatory responses [35]. Cinnamon and eucalyptus oils have good antioxidant and anti-inflammatory activities, and these two plant oils can suppress LPS-induced inflammation by reducing SOD, TNF-α, and NF-κB levels, with Cinnamon oil also increasing mouse GSH-Px, MDA, and Mn-SOD levels, as well as visceral edema coefficients in the kidneys and liver [36].

In addition, some studies have also explored the improvement of natural oils on inflammation induced by obesity [37,38]. These functional oils have a common characteristic, which is that they usually contain more unsaturated fatty acids or phenolic compounds. Increasing evidence shows that natural oils have a good role in improving inflammation.

### 3.1. The Beneficial Effects of Plant Oils on IBD

#### 3.1.1. Olive Oil

Olive oil has been consumed by humans since ancient times and has long been considered an important source of high-nutrient fat and used as a medicine to improve health [39]. There are multiple bioactive compounds present in olive oil, such as tocopherols, carotenoids, lutein, and beta-carotene, as well as a large number of phenolic compounds [40]. The anti-inflammatory activity and immune-modulating effects of olive oil have been extensively studied, and its potential preventive effects against cardiovascular diseases have been reported [41,42,43]. There are also numerous studies reporting the beneficial effects of olive oil on inflammatory bowel disease (IBD). Research has shown that the oral administration of olive oil can alleviate the elevated levels of IL-1β and oxidative damage in the colitis of SD rats, and this improvement is even stronger in genistein-enhanced olive oil [44]. In a study where 5% olive oil was used to intervene in DSS-induced colitis rats and the rats were sacrificed after continuous intervention for 5 weeks, it was found that the enhanced expression of STAT3, pSTAT3, COX-2, and iNOS induced by DSS was alleviated by olive oil, indicating that long-term intake of 5% olive oil can improve chronic inflammation in rats [45]. The beneficial effects of olive oil on colitis may require a longer duration of intake for better results. In another study, mice were given a certain amount of olive oil for 30 consecutive days before inducing colitis, and upon dissection, it was discovered that olive oil was able to improve chronic colitis by downregulating iNOS levels and enhancing the mice’s antioxidant capacity [46]. Further research has found that the non-saponifiable portion of olive oil seems to have a stronger anti-inflammatory effect. Researchers found that in an experiment evaluating the anti-colitis effects of the non-saponifiable portion of olive oil, it was able to alleviate oxidative stress and restore the expression levels of pro-inflammatory proteins to normal levels via the p38 MAPK and NF-κB signaling pathways [47].

#### 3.1.2. Perilla Oil

Perilla oil is an edible oil extracted from the seeds of *Perilla frutescens*, commonly used in daily cooking in countries such as Japan and Korea. Due to its high content of omega-3 fatty acids and various phenolic compounds, Perilla oil has multiple health benefits, such as anti-inflammatory, antioxidant, and improvement of cardiovascular diseases [48]. Numerous studies have confirmed the anti-inflammatory activity of Perilla oil [49,50]. It is worth emphasizing that Perilla oil has a particularly significant effect on improving inflammatory bowel disease. Research has found that continuous supplementation with Perilla oil for 16 weeks can significantly improve colonic inflammation induced by a high-fat diet in mice. Further exploration has found that Perilla oil can alleviate colonic inflammation by inhibiting the activation of NF-κB in the colon of mice [51]. Another study found that Perilla oil can reduce colonic inflammation in mice by protecting the intestinal barrier function, inhibiting the NF-κB pathway, and reducing the expression of pro-inflammatory genes, which may be related to the activation of GPR120 [52]. For the DSS-induced colitis model, Perilla oil also has an improving effect. Before establishing the acute colitis mouse model with DSS, mice were fed different doses of Perilla oil for three consecutive weeks. The results showed that Perilla oil pretreatment significantly improved weight loss, diarrhea, massive bleeding, and DSS-induced colonic shortening in mice, and significantly reduced the inflammatory response in colonic tissue [53]. In addition, some researchers have found that perilla oil has a protective effect on the intestinal epithelial barrier in mice with ulcerative colitis [54].

#### 3.1.3. Garlic Oil

Garlic can be used as a medicinal food and is commonly used to treat diseases such as coughs, insect bites, and constipation. Its main active ingredient comes from garlic oil [55]. Garlic oil has been used in traditional medicine to treat many inflammatory diseases, and recent studies have found that it has a relieving effect on diseases such as arthritis and bronchitis [56,57]. The anti-inflammatory effect of garlic oil is due to its excellent antioxidant, anti-inflammatory, and immune-regulating properties. Research has found that garlic oil also has a certain therapeutic effect on IBD. In an experiment on acute colitis induced by DSS in rats, researchers treated the rats with garlic oil. Garlic oil was orally administered at doses of 25, 50, and 100 mg/kg/day, and after continuous treatment for 7 days, it was found that garlic oil treatment suppressed colitis inflammation, improved colonic oxidative stress levels, and improved macroscopic and microscopic changes in the rat colonic mucosa in a dose-dependent manner [58]. Garlic oil also has a therapeutic effect on acetic acid-induced colitis inflammation. Garlic oil treatment reduced colon damage and inflammation in the acetic acid-induced colitis model, and this therapeutic effect was evident in both local and systemic treatments, with better results observed in local treatment [59].

#### 3.1.4. Flaxseed Oil

Flaxseed oil is an oil extracted from the seeds of the flax plant (*Linum usitatissimum* L.) and is widely produced worldwide with a considerable yield. It is commonly used in the production of baked goods such as cakes, bread, and cookies [60,61]. Flaxseed oil is recognized as one of the foods rich in alpha-linolenic acid, and a large body of research has confirmed the ability of ALA to alleviate colon inflammation in DSS-induced colitis in rats [62,63]. Researchers administered flaxseed oil at doses of 400, 800, and 1600 mg/kg bw to rats via gavage for six weeks and induced ulcerative colitis with 3% DSS daily during the sixth week. The results showed that long-term consumption of flaxseed oil significantly improved the pathological manifestations of colitis in rats, and partially restored DSS-induced alterations in gut microbiota [64]. Similarly, the beneficial effect of flaxseed oil on colitis was also demonstrated in a mouse experiment. In mice with acetic acid-induced colitis, flaxseed oil was able to improve colitis through its antibacterial and antioxidant activities and had a reversing effect on immune dysregulation, microbial dysbiosis, and intestinal barrier damage in colitis mice [65].

#### 3.1.5. Walnut Oil

Walnut oil is a high-quality nut oil that is rich in unsaturated fatty acids such as linoleic acid and alpha-linolenic acid, and is considered a healthy premium edible oil [66,67]. Walnut oil has been used in traditional medicine, and recent research has found that it has anti-inflammatory effects by inhibiting inflammatory factors and increasing antioxidant capacity [68,69]. Walnut oil also has a beneficial effect on IBD. It is reported that walnut oil can alleviate colonic inflammation by regulating the expression of tight junction proteins, free fatty acid receptors, and pro-inflammatory cytokine gene proteins, and can protect the intestinal barrier function of colitis mice [70]. The latest research also shows that walnut oil improves colitis symptoms in mice by inhibiting NLRP3 inflammasome activation and regulating intestinal microbiota [71]. The therapeutic effect of walnut oil on colitis may serve as a source of raw materials for functional foods that alleviate inflammation.

#### 3.1.6. Yadanzi Oil

*Brucea javanica* (L.) Merr. is a traditional Chinese medicinal herb that has been used to treat dysentery, malaria, cancer, and other diseases in China and Southeast Asian countries [72,73]. Yadanzi oil is extracted from the mature fruits of *Brucea javanica* and possesses multiple biological activities, serving as the main source of functional activity for *Brucea javanica* [74]. Due to the presence of multiple active compounds, Yadanzi oil exhibits strong anti-inflammatory effects [75]. Studies have shown that Yadanzi oil has therapeutic effects on DSS-induced colitis in mice, as it can suppress the NF-κB signaling pathway and downregulate inflammatory mediators to exert anti-inflammatory effects [76]. A recent study also demonstrated that Yadanzi oil enriched with brusatol can simultaneously inhibit both NF-κB and RhoA/ROCK signaling pathways and restore intestinal barrier function to improve mice with ulcerative colitis [77]. This finding also confirms that brusatol is one of the active compounds in Yadanzi oil for treating UC.

#### 3.1.7. Grape Seed Oil

Grape seed contains 8–20% oil, with varying amounts of phenolic compounds, flavonoids, and unsaturated fatty acids depending on the grape variety [78]. Active substances in grape seed oil have been shown to have multiple pharmacological activities [79]. The grape seed oil has therapeutic effects on various models of IBD. For example, a study found that *Vitis vinifera* (black grape) seed oil had a protective effect on acetic acid-induced colitis in rats, improving their oxidative stress levels [80]. Another study found that administering grape seed oil enema significantly improved inflammation in colitis rats [81]. In addition, grape seed oil rich in Carbon 60 significantly reduced the inflammatory response in DSS-induced colitis rats [82]. The therapeutic effect of grape seed oil on IBD is attributed to its powerful antioxidant activity.

In Table 1, we provided a comprehensive summary of the beneficial effects and mechanisms of action of plant oils in IBD.

### 3.2. Improvement Effect of Animal Oil on Experimental IBD

The oils that have a therapeutic effect on experimental IBD are mainly derived from plants, but research has found that several animal-derived oils also exhibit excellent anti-inflammatory activity, such as emu oil, fish oil, and yellow mealworm larva oil (Table 2).

Emu oil is an animal oil extracted from the peritoneum and subcutaneous fat of the bird species *Dromaius novaehollandiae*. Indigenous Australians were the first to use emu oil to treat arthritis and promote wound healing [102]. Emu oil is rich in a variety of unsaturated fatty acids, including oleic acid, linoleic acid, alpha-linolenic acid, and palmitoleic acid, with a content of up to 50% [103]. Some studies have found that emu oil has anti-inflammatory effects on arthritis and gastroenteritis [102,104]. Importantly, numerous studies have also demonstrated the good therapeutic effects of emu oil on IBD. In a Crohn’s disease model in rats, emu oil improved intestinal inflammation by inhibiting oxidation and improving histological changes, and this effect was even better when used in combination with aloe vera [105]. In addition, another study found that emu oil could alleviate the overall disease severity and facial expression scores of Crohn’s disease mice, indicating its huge therapeutic potential in controlling Crohn’s disease when taken orally [106]. In a mouse model of acetic acid-induced ulcerative colitis, emu oil also has a therapeutic effect. The study found that emu oil significantly improved the inflammation level of ulcerative colitis mice, and when used in combination with glycyrrhizin, it showed a stronger regulatory effect on the expression of PPARγ and TNFα, with a synergistic effect on the regulation of PPARγ and TNFα expression [107]. In a chronic colitis model induced by DSS, the oral administration of emu oil significantly reduced the severity of clinical and histological evaluations of colitis mice [108]. Emu oil has a significant improvement effect on colitis-related tissue damage, indicating its potential as a candidate drug for the treatment of IBD [109].

In addition, yellow mealworm larva oil has also been found to have an improving effect on ulcerative colitis. After the administration of yellow mealworms larva oil in a mouse model of DSS-induced ulcerative colitis, the colon length of colitis mice increased, spleen weight decreased, body weight increased, disease activity index levels decreased, and colitis inflammatory cytokine levels decreased. This improvement is related to the regulation of the NF-κB signaling pathway by yellow mealworms larva oil [110]. Furthermore, fish oil can also improve weight loss and colon bleeding in DSS-induced colitis mice [111]. The common feature of these animal oils is that they are rich in unsaturated fatty acids, which give them strong antioxidant activity and can alleviate IBD.

**Table 2:** Improvement of experimental IBD by animal oils.

Oil	Source	Composition	Dosage	Model	Mechanism	Year	Reference
Yellow mealworm larva oil	*Tenebrio molitor*	Rich in oleic acid (45%), linoleic acid (20%), and polyunsaturated fatty acids (20%).	50, 100 µL oral administration	DSS-induced colitis in ICR mice	Regulating the NF-κB signaling pathway to reduce inflammation.	2022	[110]
Fish oil	/	Rich in omega-3 fatty acids.	10%	DSS-induced colitis in mice	Slowing down weight loss and colon bleeding.	2013	[111]
Emu oil	*Dromaius novaehollandiae*	Rich in oleic acid (42%), linoleic acid (21%), and palmitic acid (21%).	80, 160 µL oral administration	DSS-induced chronic colitis in C57BL/6 mice	Reducing the severity of clinical and histological disease.	2019	[108]
		Rich in oleic acid (42%), linoleic acid (21%), and palmitic acid (21%).	0.5, 1 mL	DSS-induced colitis in SD rats	Improved tissue damage associated with colitis.	2012	[109]
		Rich in docosahexaenoic acid and eicosapentaenoic acid.	10 mL/kg	Acetic acid-induced colitis in Wistar rats	Regulating the expression of PPARγ and TNF-α.	2015	[107]
		Rich in oleic acid (36.4%), linoleic acid (8%), and palmitic acid (6.3%).	80, 160 µL by gavage	TNBS-induced colitis in ARC(s) mice	Reducing the severity of colitis and facial grimace scores.	2020	[106]
		Rich in oleic acid, linoleic acid, linolenic acid, and palmitoleic acid.	10 mL/kg BW	Indomethacin-induced colitis in Wistar albino rats	Inhibiting oxidation and improving colonic histological morphology.	2016	[105]

“/” indicates not stated in the literature.

## 4. The Clinical Improvement of Natural Oils in IBD

Currently, several commonly used experimental IBD models provide opportunities to explore new drugs that may improve intestinal inflammation. However, to date, no animal model can fully reflect the complexity of human IBD, so validation results from animal experiments may not be entirely applicable to humans. Therefore, a compound can only be considered a potential candidate for the treatment of human IBD if it achieves good results in multiple experimental IBD models [112]. Therefore, the therapeutic effect of natural oils on IBD cannot be concluded solely from animal models, and extensive clinical trials are needed before they can be used as drugs to treat diseases. Currently, a large amount of research on the therapeutic effects of natural oils on IBD is still limited to animal models, but in some clinical trials, natural oils have also shown improvement in human IBD (Table 3).

In a double-blind clinical trial, 38 IBD patients (21 with CD and 17 with UC) were given 10 mL of seal oil orally every day for 14 days. The results showed no significant differences between CD and UC patients, but the short-term use of seal oil improved IBD-related joint pain [113]. Other clinical trials have found that seal oil can alter the fatty acid composition and n-3/n-6 ratio in the blood and intestinal mucosa of IBD patients [114]. In addition, in a randomized controlled clinical trial, 90 UC patients were treated with flaxseed oil for 12 weeks. After the treatment ended, UC indicators were evaluated, and it was found that flaxseed oil could alleviate inflammation markers, improve the severity of UC, and metabolic parameters [115]. Another crossover clinical trial investigated the therapeutic effect of extra virgin olive oil on UC patients and found that ingesting olive oil can reduce inflammation markers in UC patients and improve gastrointestinal symptoms, indicating that olive oil may be used as a functional food to assist in the treatment of UC with medication [116]. In addition, a successful case of using coconut oil for rectal enema treatment in a patient with metastatic colitis has been reported [117].

**Table 3:** Clinical improvement effects of natural oils on IBD.

Oil	Composition	Patients	Treatment Method	Effect	Year	References
Seal oil	Rich in n-3 fatty acids.	21 had CD and 17 UC	Take 10 mL seal oil orally 3 times a day for 14 days.	Altering the fatty acid composition and n-3/n-6 ratio in blood and intestinal mucosa, improving disease activity and trend of IBD-related joint pain.	2008	[113,114]
Coconut oil	Rich in short-chain fatty acids.	DiversionColitis	100 mL of pre-warmed coconut oil was given topically daily as a rectal enema.	After one week of treatment, abdominal pain and mucus secretion decreased. After six weeks, there was a significant improvement in inflammatory and histological symptoms. After twelve weeks, the patient had recovered.	2018	[117]
Extra virgin olive oil	70.9% oleic acid, 9.7% linoleic acid, 14.8% palmitic acid, 2.1% stearic acid, and 0.5% alpha-linolenic acid.	40 patients with UC	Take 50 mL orally with meals daily for 20 days.	After intervention, both erythrocyte sedimentation rate and high-sensitivity C-reactive protein were significantly reduced. Symptoms such as abdominal distension, constipation, fecal urgency, and incomplete bowel movements were alleviated.	2020	[116]
Flaxseed oil	Rich in omega-3 fatty acids, phytoestrogens, and soluble fiber.	90 patients with UC	10 g daily for 10 weeks.	It can significantly reduce inflammation markers, disease severity, blood pressure, and urine output in patients with colitis.	2019	[115]
*Evening primrose* oil	Rich in GLA (15.5%).	43 patients with UC	Take 12 capsules per day for 1 month, then 6 capsules per day for 5 months (each capsule contains *evening primrose* oil 250 mg).	The red blood cell membrane concentration of dihomo-gamma-linolenic acid (DGLA) increased by 40%.	1993	[118]

## 5. Main Pathways of Action

**Figure 2:**
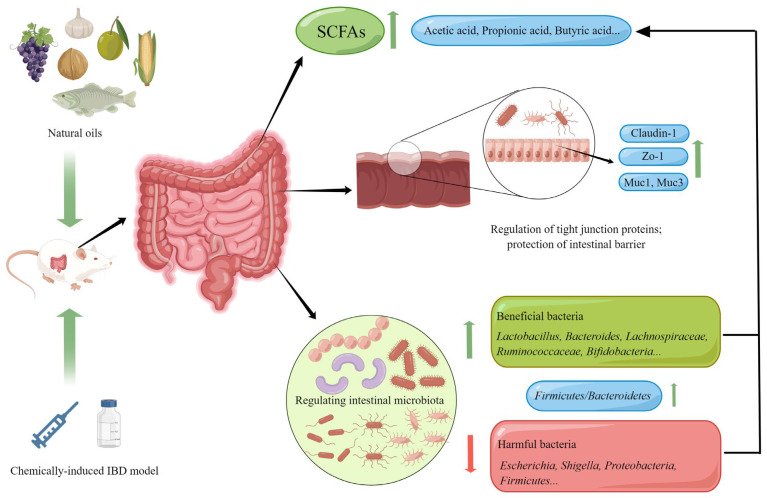
Natural oils regulate the intestinal microbiota and improve the intestinal barrier mechanism (By Figdraw.).

Natural oils can improve intestinal homeostasis in IBD animal models through various pathways, including regulating the gut microbiota, protecting the intestinal barrier, reducing colonic inflammation levels, improving oxidative stress levels in the gut, and regulating immune homeostasis. Figure 2 illustrates the mechanism by which natural oils regulate the gut microbiota and improve the intestinal barrier.

### 5.1. Regulating Intestinal Flora

The gut microbiota refers to all microbial communities in the gut, including bacteria, archaea, fungi, and viruses. The relative composition and the abundance of gut microbiota have significant impacts on the host’s health. In recent years, the stability of gut microbiota has become one of the hot topics in the study of IBD. Many studies have confirmed the close relationship between the occurrence and development of IBD and gut microbiota [119]. IBD patients often suffer from gut microbiota dysbiosis, including a reduction in microbial and metabolite diversity, an increase in pathogenic bacteria, and a decrease in beneficial bacteria [120]. Research has found that some natural oils have a regulatory effect on the gut microbiota of IBD patients. For example, flaxseed oil can restore the changes in the cecum microbiota of DSS-induced rats. The results showed that the oil increased the abundance of *Firmicutes/Bacteroidetes* and to some extent increased the abundance of *Lactobacillus*, *Lachnoclostridium*, *Lachnospiraceae_NK4A136_group*, and *Ruminococcaceae_UCG-005* [64]. Walnut oil not only increased the relative abundance of probiotics (*Lactobacillus*, *Lachnospiraceae_NK4A136_group*, *Faecalibaculum*, *Bifidobacterium*, *Akkermansia*, and *Roseburia*), but also reduced the relative abundance of pathogenic bacteria, and increased the content of short-chain fatty acids such as acetic acid, propionic acid, and butyric acid in the gut of UC mice [71]. Similarly, the intake of Camellia oil can also alter the gut microbiota of acetic acid-induced colitis mice, increasing the ratio of *Firmicutes/Bacteroidetes*, alpha diversity, and the relative abundance of *Bifidobacterium* [84]. In addition, algal oil can also regulate the gut microbiota in UC [87].

### 5.2. Protecting the Intestinal Barrier

The gut epithelium and the covering mucus layer together form the intestinal barrier. In patients with IBD, the intestinal barrier is disrupted, as shown by a decrease in goblet cells, a defect in defense molecule generation, mucosal barrier damage, and changes in mucosal composition as observed under a microscope [121]. Intestinal barrier integrity is crucial for gut health, and a disrupted intestinal barrier exacerbates colonic inflammation in IBD patients [122]. Natural oils have a protective effect on the intestinal barrier. Supplementation with perilla oil has been found to significantly improve goblet cells that have been damaged by DSS, protect the integrity of the gut epithelium, and increase the expression levels of markers of epithelial barrier integrity, such as Claudin-1, Zo-1, Muc1, and Muc3, as shown by western blot analysis in colonic tissue [52]. Walnut oil also has a protective effect on intestinal barrier function. After the ingestion of walnut oil, the expression levels of tight junction (TJ)-related proteins in colonic tissue increased in colitis mice, and walnut oil reversed the loss of TJ-related proteins induced by inflammation [70]. Flaxseed oil, algal oil, and angelica oil have also been found to have a protective effect on the intestinal barrier [65,87,91]. This indicates that restoring intestinal barrier function is one of the targets of natural oils in improving IBD.

### 5.3. Reduces Inflammatory Response

IBD patients often experience inflammation in their gastrointestinal tract, and natural oils can alleviate intestinal inflammation through various pathways, thereby improving IBD symptoms. Most studies on the anti-IBD effects of natural oils have evaluated the regulatory effects of oils on inflammation-related signaling pathways, including NF-κB, MAPK, and PPARγ signaling pathways. NF-κB is a transcription factor that promotes the expression of pro-inflammatory cytokine genes. In IBD patients, NF-κB signaling is often dysregulated, leading to excessive inflammation [123]. The activation of the MAPK and PPARγ signaling pathways is also associated with the degree of inflammation in IBD patients [124]. In a high-fat diet-induced colitis mouse model, perilla oil reduced the levels of pro-inflammatory cytokines in the serum and colon and reduced the severity of colitis in mice by inhibiting the activation of NF-κB in the colon [51,52]. Similarly, yadanzi oil has a significant anti-inflammatory effect on DSS-induced UC mice, and this protective effect is related to the inhibition of the NF-κB signaling pathway and subsequent downregulation of inflammatory mediators [76]. Studies have also found that yadanzi oil can not only inhibit the NF-κB signaling pathway but also the RhoA/ROCK signaling pathway in UC mice [77]. Yellow mealworm larva oil also has the function of inhibiting the NF-κB signaling pathway in UC mice [110]. In addition, studies have found that extra-virgin olive oil can alleviate acute colitis in mice through the MAPK and NF-κB signaling pathways [47]. Yarrow oil can also alleviate the degree of inflammation and regulate the secretion of inflammatory cytokines by regulating the NF-κB and PPAR-γ pathways in a UC mouse model [96]. The above research results confirm the anti-inflammatory activity of natural oils in experimental IBD models.

### 5.4. Improve Oxidative Stress Levels

Oxidative stress, often accompanied by inflammation, is also considered one of the important factors in the development and progression of IBD [125]. IBD leads to increased production of free radicals, such as superoxide anion, hydrogen peroxide, and hydroxyl radicals, resulting in elevated oxidative stress levels in IBD patients [126]. The active ingredients in camellia oils have strong antioxidant activity, and when colitis rats were given a certain amount of Camellia oils, the levels of MDA in their colon tissues decreased, while SOD activity and GSH levels increased, indicating that the antioxidant activity of camellia oils can improve the oxidative stress level in colitis rats [85]. Similarly, when UC rats were orally administered flaxseed oil for six consecutive weeks at a dose of 1600 mg/kg BW, the oxidative stress level in their colon tissues was significantly reduced, as evidenced by increased SOD activity and GSH levels, as well as decreased MPO activity and MDA levels [64]. In addition, studies have shown that natural oils such as rice bran oil, garden cress (*Lepidium sativum*) seed oil [98], *Vitis vinifera* (black grape) seed oil [80], and copaiba oil [101] can also reduce the oxidative stress levels in the colon tissues of IBD mouse models.

### 5.5. Regulation of Intestinal Immune Homeostasis

In the pathophysiology of IBD patients, immune dysregulation is often accompanied, and the occurrence, development, and migration of inflammation in the body are related to innate and adaptive immunity. Some natural oils can help treat IBD by affecting the release of immune-related cytokines and the immune homeostasis of the intestinal mucosa. TLR2 is a recognition receptor expressed in colonic epithelial cells and innate immune cells, which can enhance the production of pro-inflammatory cytokines and immune regulatory factors [127]. Garlic oil can inhibit inflammation and promote healing by inhibiting TLR2 activation, owing to its immunomodulatory properties [58]. Studies have also shown that the intake of pequi oil helps regulate the immune homeostasis of the colons of UC mice, as evidenced by an increase in γδT cells and secretory IgA and a decrease in CD8+T cells [86]. It is worth noting that the pathogenesis of IBD is also associated with the excessive activation of Th17 cells and overexpression of IL-17 and IL-23 [128]. However, natural oils such as *Brucea javanica* oil [76] and cottonseed oil [97] can effectively improve the Th17/Treg immune imbalance by reducing the overexpression of IL-17. These studies provide new insights into the immunomodulatory role of natural oils in IBD.

## 6. Conclusions

Oils from different plants and animals have been validated in numerous experimental IBD animal models, and these oils can independently maintain, enhance, and improve intestinal homeostasis in IBD animal models through multiple mechanisms, including regulating gut microbiota, protecting intestinal barrier, reducing colonic inflammation levels, improving oxidative stress levels in the gut, and regulating immune homeostasis. Moreover, the IBD therapeutic effects of some natural oils have entered the clinical stage and achieved certain results. However, these works are still in their infancy and require further evaluation in more IBD patients to explore their mechanisms of action, optimal administration routes, and best doses. Additionally, given that mixed oils typically contain various types of fatty acids, future research should focus on the specific fatty acids present in natural oils that contribute to the improvement of IBD, such as long-chain fatty acids [129] and n-3 polyunsaturated fatty acids [130,131]. These specific fatty acids play an important role in regulating intestinal inflammation and immune homeostasis in IBD patients. Ongoing and future research may provide more information on the beneficial effects of natural oils on human IBD and provide a basis for further developing functional products or drugs based on natural oils for IBD.

## Figures and Tables

**Table 1:** Improvement effects of plant oils on experimental IBD.

Oil	Source	Composition	Dosage	Model	Mechanism	Year	Reference
Lentisc oil	*Pistacia lentiscus*	Oleic acid (47.96%), palmitic acid (27.94%), and linoleic acid (20.22%).	30 mg oil/100 g of food/rat	TNBS-induced colitis in Wistar rats	Modification of arachidonic acid metabolism.	2016	[83]
Camellia oil	*Camellia oleifera* Abel.	Rich in oleic acid, linoleic acid, and palmitic acid.	2 mL/kg BW	Acetic acid-induced colitis in SD rats	Modulation of gut microbiota; significant reduction in inflammatory injury and lipid peroxidation.	2018	[84]
		/	2 mL/kg BW	Acetic acid-induced colitis in SD rats	Modulation of gut microbiota, reduction in oxidative stress, and inhibition of inflammatory responses.	2020	[85]
Flaxseed oil	*Linum usitatissimum* L.	Rich in omega-3 fatty acids.	400, 800 and 1600 mg/kg BW	DSS-induced colitis in SD rats	Modulation of oxidative conditions, inflammatory factors, and cecal microbiota.	2020	[64]
			100, 300 and 500 mg/kg BW	Acetic acid-induced colitis in SD rats	Improvement of immune dysregulation, barrier defect, and microbial imbalance in colitis rats.	2020	[65]
Garlic oil	*Allium sativum*	/	25, 50 and 100 mg/kg/day	DSS-induced colitis in Wistar rats	Improvement of colitis in rats through its antioxidant, anti-inflammatory, and immunomodulatory properties.	2016	[58]
		/	5 mL/kg	Acetic acid-induced colitis in Wistar rats	Alleviation of colon damage and inflammatory response in a model of acetic acid-induced colitis.	2020	[59]
Perilla oil	*Perilla frutescens*	Rich in omega-3 fatty acids (ranging from 54% to 60%).	Supplementation with perilla oil for 16 weeks.	High-fat diet-induced inflammation in the colon of C57BL/6J mice	Inhibition of the activation of the NF-κB pathway.	2020	[51]
		Rich in omega-3 fatty acids (59.7%).	8%	High-fat diet-induced inflammation in the colon of C57BL/6J mice	Protecting the intestinal barrier, inhibiting NF-κB pathway, reducing the expression of pro-inflammatory genes.	2022	[52]
		Rich in alpha-linolenic acid (61.51%).	20, 100, 200 mg/kg BW	DSS-induced colitis in C57BL/6 mice	Significantly reducing inflammation in colon tissues.	2021	[53]
		Rich in polyunsaturated fatty acids.	8%	High-fat diet-induced inflammation in the colon of C57BL/6J mice	Significantly reducing levels of inflammatory factors in both the serum and colon.	2022	[54]
Pequi oil	*Caryocar* *brasiliense*	Rich in oleic acid and carotenoids.	280 mg of pequi oil was homogenized in 1.1 g of chow.	DSS-induced colitis in C57BL/6 mice	Impact on the intestinal immune system and improvement of DSS-induced intestinal immune injury.	2021	[86]
Grape seed oil	/	/	1 mL enema therapy.	Acetic acid-induced colitis in SD rats	The antioxidant properties of grape seed oil improve colitis.	2017	[81]
		Rich in Carbon 60.	1 mL/kg	DSS-induced colitis in Wistar rats	Improvement of clinical symptoms in rats with colitis.	2022	[82]
Algal oil	/	Rich in docosahexaenoic acid.	250 or 500 mg/kg/day	DSS-induced colitis in C57BL/6 mice	Modulating the gut microbiota and metabolites to restore the intestinal barrier.	2020	[87]
Walnut oil	/	Rich in linoleic acid and alpha-linolenic acid.	2.5 mL/kg/day	DSS-induced colitis in C57BL/6 mice	Inhibiting the activation of NLRP3 inflammasome and modulating the gut microbiota.	2021	[71]
		Rich in linoleic (55–70%) and α-linolenic (10–18%) acids.	7%	DSS-induced colitis in C57BL/6 mice	Alleviating mouse intestinal inflammation and restoring intestinal barrier function.	2020	[70]
Yadanzi oil	*Brucea javanica*	Rich in hexadecanoic acid, linoleic acid, oleic acid.	0.5, 1 and 2 g/kg	DSS-induced colitis in Balb/C mice	Inhibition of NF-κB activation.	2017	[76]
		Rich in brusatol, oleic acid and fatty acids.	152.5, 305, 610 mg/kg/day	DSS-induced colitis in Balb/C mice	Inhibition of NF-κB and RhoA/ROCK signaling pathways.	2021	[77]
Stinging nettle seed oil	*Urtica dioica*	Rich in palmitic acid, oleic acid, and linoleic acid.	2.5 mL/kg	TNBS-induced colitis in Wistar rats	Improving colitis through its anti-inflammatory and antioxidant effects.	2011	[88]
Cinnamon oil	*Cinnamomum zeylanicum*	/	630, 1250, 2500, 5000 ppm	TNBS-induced colitis in ICR rats	Modulating intestinal bacteria to improve intestinal wall injury.	2013	[89]
Menthacarin	*Mentha piperita* L.; *Carum carvi* L.	Peppermint oil and caraway oil	1, 3, 6, and 12 µg/µL	DSS-induced colitis in C57BL/6 mice	Improving clinical symptoms of colitis in mice through its anti-inflammatory effect.	2020	[90]
Angelica oil	*Angelica sinensis*	Rich in ligustilide and linoleic acid.	10, 20, and 40 mg/kg	DSS-induced colitis in C57BL/6J mice	Restoring intestinal barrier function by inhibiting the S100A8/A9 signaling pathway.	2023	[91]
Ozonized olive oil	/	Rich in oxygenated triglyceride.	3 and 6 mg/kg	DNBS-induced colitis in albino rats	CAT, GSH-Px, and SOD activities in the colon were significantly increased in a dose-dependent manner compared to the control group.	2014	[92]
Black cumin oil	*Nigella* *sativa*	Rich in linoleic and oleic acids.	2.5 mL/kg	TNBS-induced colitis in Wistar albino rats	Reduced pro-inflammatory cytokines, lactate dehydrogenase, triglycerides, and cholesterol.	2011	[93]
Olive oil	/	Rich in EPA and DHA polyunsaturated fatty acids.	4%	DSS-induced colitis in Wistar rats	Reduced colonic inflammation.	2005	[94]
Extra virgin olive oil	/	/	5 mL/Kg BW	Acetic acid-induced colitis in SD rats	Alleviated the elevated levels of IL-1β and oxidative damage in colitis.	2020	[44]
		/	5%	DSS-induced colitis in SD rats	Reduced the expression of STAT3, pSTAT3, COX-2, and iNOS in colitic rats, inhibiting chronic inflammation.	2014	[45]
		Enriched with hydroxytyrosol.	40 mg/kg	DSS-induced colitis in C57BL/6 mice	Improving colitis by modulating the cytokines COX-2 and iNOS.	2012	[46]
		Unsaponifiable.	/	DSS-induced colitis in C57BL/6 mice	Improving colitis by modulating the p38 MAPK and NFκB signaling pathways.	2013	[47]
Pomegranate seed oil	*Punica granatum*	Rich in conjugated linolenic acids, oleic acid, linoleic acid.	1.5%	Necrotizing enterocolitis in SD rats	Improving intestinal epithelial homeostasis and mucosal inflammation.	2012	[95]
Black grape oil	*Vitis vinifera*	Rich in unsaturated fatty acids.	2, 4, and 8 mL/kg	Acetic acid-induced colitis in Wistar rats	Improving oxidative stress.	2020	[80]
Yarrow oil	*Achillea millefolium*	Rich in sabinene and pinene.	100 mg/kg	DSS-induced colitis in C57BL/6 mice	Mitigating UC symptoms and regulating the secretion of inflammatory cytokines by modulating the NF-κB and PPAR-γ pathways.	2021	[96]
Cottonseed oil	/	/	200 μL/day	DSS-induced colitis in C57BL/6 mice	Improving intestinal inflammation by reducing inflammatory cytokines and oxidative stress markers.	2019	[97]
Garden cress seed oil	*Lepidium sativum*	Rich in n-3 fatty acid.	10%	DSS-induced colitis in Wistar rats	Reduce oxidative damage, inhibit inflammatory mediators, and decrease damage to the colon.	2014	[98]
Rice bran oil	/	Rich in oryzanol (1.2%).	10%	DSS-induced colitis in Wistar rats	Reduced oxidative damage, inhibition of inflammatory mediators, and decreased damage to the colon.	2014	[98]
Ulvaceae oil	*Ulva ohnoi*	/	25 mg/kg	DSS-induced colitis in ICR mice	Reduced expression of inflammatory cytokines.	2021	[99]
Mandarin oil	/	/	200, 400 mg/kg	Indomethacin-induced colitis in Wistar rats	Exhibit antioxidant and anti-inflammatory effects against rat enterocolitis.	2014	[100]
Copaiba oil	*Copaifera reticulata*	Rich in β-caryophyllene (37.6%), β-bisabolene (13.9%).	1.15 g/kg	TNBS-induced colitis in Wistar rats	Reduce oxidative stress and inflammation.	2018	[101]

“/” indicates not stated in the literature.

## Data Availability

Not applicable.
